# Acute onset anarthria in a 7-year-old patient as a presentation of acute disseminated encephalomyelitis: A rare clinical and radiological entity

**DOI:** 10.1016/j.radcr.2021.08.016

**Published:** 2021-09-04

**Authors:** Rachid Belfkih, Omar Ghomari Khayat

**Affiliations:** a Abdelmalek Saadi University, Faculty of medicine and pharmacy of Tangier, Tangier, Morocco; b Department of neurology, University Hospital Center of Tangier-Tetouan-Al hoceima, Tangier, Morocco

**Keywords:** Case report, Acute disseminated encephalomyelitis, Multiple sclerosis, Anti-myelin oligodendrocyte glycoprotein (anti-MOG) antibodies, Corticosteroids

## Abstract

Acute disseminated encephalomyelitis (ADEM), also called post-infectious encephalomyelitis, is defined as an immune-mediated inflammatory and demyelinating event that involves the central nervous system. It usually follows an infectious episode or an active immunization several weeks prior the disease onset. ADEM is typically presented with an encephalopathy associated to focal neurological signs. Cerebrospinal fluid analysis is usually nonspecific, showing signs of central nervous system inflammation with negative viral and bacterial cultures. The diagnosis is based on clinical and MRI findings. Patients with ADEM respond well to anti-inflammatory and immunosuppressive agents, with high-dose of intravenous steroids administered as first-line treatment. Herein, we present the case of a 7-year-old male patient diagnosed of acute disseminated encephalomyelitis most likely secondary to a viral upper respiratory tract infection. It is a unique case, as the inaugurating sign is an acute onset anarthria. The patient responded favorably to the first-line therapy with an almost full recovery within the first week of treatment.

## Introduction

Acute disseminated encephalomyelitis (ADEM) is a demyelinating disease affecting the central nervous system (CNS). Typically presented in childhood, the mean age of its occurrence ranges between 5 and 8 years, with male predominance [1]. At medical history evaluation, a recent infectious illness or vaccination is signaled in about 75% of pediatric cases [2]. Most infectious agents associated with ADEM are viruses *(Coronavirus, Coxsackie B, dengue, hepatitis A virus, hepatitis C virus, herpes simplex virus, varicella zoster virus, Epstein-Barr virus, human herpesvirus-6, human immunodeficiency virus, measles, mumps, rubella, and parainfluenza*), followed by bacteria *(Streptococcus, Mycoplasma, Legionella, Chlamydia, Borrelia, Rickettsia, Campylobacter)* and parasites *(Plasmodium vivax, Toxoplasma gondii)* which are less commonly involved [3]. The time interval between the febrile event and the onset of the neurological symptoms varies from 2-30 days [4]. We hereby present a clinical case of a 7-year-old male patient diagnosed with ADEM.

## Clinical presentation

A 7-year-old male patient presented to the pediatric emergency room with acute suspension of speech (inability to articulate speech, defined as anarthria) with mildly open and stiff mouth, and feverish lethargy. Prior to the onset of his condition, he hasn't submitted any complaints of headaches, blurred vision, increased sensitivity to light, nausea or vomiting to his surroundings. There was a medical history of viral tonsillitis in the previous 2 weeks, with no recent vaccination. His psychomotor development is normal, and no significant family history was reported.

On physical exam, vital parameters such as pulse, respiratory rate and blood pressure were within standard values, while his body temperature was reaching 37.9°C. On neurological exam, the patient was conscious and responsive with preserved comprehension. He presented a neck stiffness with negative Kernig and Brudzinski signs. No sensory or motor deficits were detected, and no ataxia or any other gait or stance abnormalities were observed. His muscle strength in lower and upper limbs was 5/5 on the Medical Research Council scale. During trigeminal nerve examination, there was a rigidity of masseter muscles with an inability of opening or moving the jaw, the rest of cranial nerves exam was normal. Deep tendons reflexes were brisk with normal plantar reflexes. There was no tremor or other involuntary movements. Coordination exam was likewise normal. The rest of cardio-vascular, respiratory, abdominal and dermatological exams revealed no abnormality.

Investigations included a blood count and C-reactive protein test that were within normal range. Brain computerized tomography scan did not disclose any significant input. A spinal tap was performed to analyze cerebrospinal fluid (CSF), and the patient was put on an empiric antibiotic treatment (third generation cephalosporin) and an antiviral drug (Acyclovir). CSF analysis was negative and did not reveal any sign of inflammation (white blood cell count was at 5 cells/mL, glucose at 28 mg/100 mL and protein at 79 mg/100 mL). Antibiotics were interrupted while the antiviral drug was continued.

**Fig. 1:**
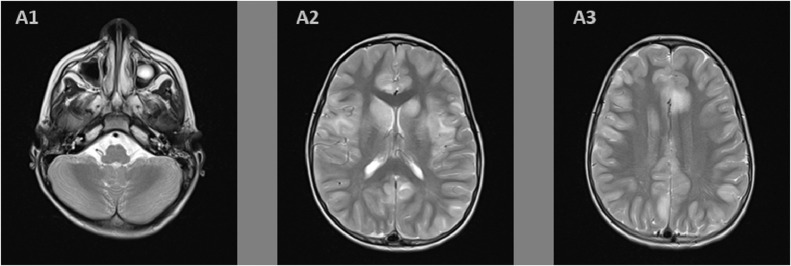
T2 weighted sequence axial images showing in the supratentorial region (A2, A3) hyperintense signals abnormalities, with nodular shape and poorly defined borders, involving bilateral central white matter, cortical gray matter, and basal ganglia. In the infratentorial region (A1), we find normo-intense signals of cerebellar and mesencephalon parenchyma, with normal fourth ventricle caliber.

**Fig. 2:**
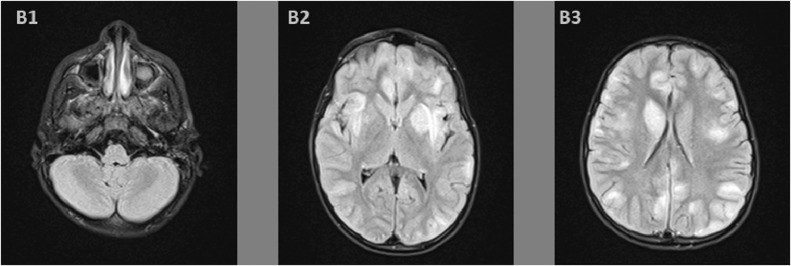
Fluid attenuated inversion recovery (FLAIR) sequence, the axial supratentorial images (B2, B3) show increased signal abnormalities, with nodular shape and poorly defined borders, involving bilateral central white matter, cortical gray matter, and basal ganglia. Infatentorial image (B1) show normal intense signal of cerebellar and mesencephalon parenchyma, with normal fourth ventricle caliber.

**Fig. 3:**
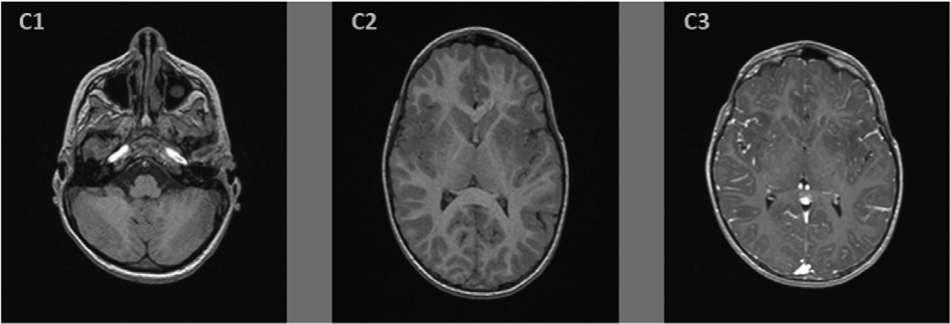
Pre-gadolinium T1 weighted sequence axial images (C1, C2) show normal signals in the infratentorial and supratentorial regions. Post-gadolinium T1 weighted sequence show no enhancement abnormalities (C3).

On the second day of admission, the anarthria was still persisting, the patient became obtunded and developed rigidity in all 4 limbs, and a quadriparesis with a Medical Research Council muscle strength scale of 3, only ocular movements were preserved, which resembled to a partial Locked-in syndrome. On that day, a brain magnetic resonance imaging (MRI) was performed, it showed a supratentorial asymmetrical increased signals in both T2-weighted and fluid attenuated inversion recovery sequences (Figs. 1 and 2), describing ovoid shape lesions with poorly discerned margins, involving bilateral central white matter, cortical gray matter, and basal ganglia. In pre- and post-contrast T1-wheighted sequences (Fig. 3), no apparent signal changes or lesion enhancement were detected.

Based on the lesions revealed on brain MRI and the clinical presentation, 3 out of 4 criteria of the revised International Pediatric Multiple Sclerosis Study Group (IPMSSG) criteria for pediatric ADEM diagnosis have been met. Hence, the diagnosis of acute disseminated encephalomyelitis was highly probable.

The patient was started on intravenous corticosteroids (methylprednisolone) at the dose of 20mg/kg/d, with continued antiviral treatment at the dose of 20 mg/kg/8 h for persistent suspicion of viral acute encephalitis.

The day following the first administration of corticosteroids perfusion, the patient anarthria have started to resolve. Rigidity and muscle weakness improved significantly within the first week of treatment. Hereafter, the patient was discharged and put on oral prednisone with a starting dose of 2 mg/kg/d during the first week, and tapered over the period of the 5 following weeks.

At 3 and 6 months clinical follow-ups, neither the patient nor his family have brought forward the onset of new neurological symptoms during this time interval, and physical exams were all normal. Thence, the patient had met all IPMSSG criteria for pediatric ADEM diagnosis.

## Discussion

Acute disseminated encephalomyelitis presents clinically with an acute onset encephalopathy and focal neurological deficits. The latter is generally preceded, within 2-5 days, by prodromal symptoms such as fever, irritability, malaise, headache, somnolence, nausea and vomiting [1]. Encephalopathy include behavioral changes and consciousness alteration, its severity varies from lethargy to coma. It is often associated to headache, seizures, vomiting, and signs of meningismus. The presence of severe signs of encephalopathy in children serves to distinguish ADEM diagnosis from multiple sclerosis (MS) due to gray matter involvement, as the cerebral cortex is responsible of sensory functions that explains the onset of deeper states of encephalopathy. Neurological signs include, depending on which brain area is involved; hemiparesis and eventually hemiplegia with possible association to pyramidal signs (Babinski sign, spasticity, hyperreflexia), ataxia, speech impairment, diplopia, homonymous hemianopia, and if severe and bilateral damage were to reach the occipital lobes, cortical blindness [4]. Brainstem damage is usually associated with poor prognosis due to the risk of respiratory failure occurrence [1]. Thus, our clinical case is unusual as ADEM rarely presents by an anarthria with no associated other neurological deficits, which could be misleading at first encounter. Only its evolution on the following days by the onset of an encephalopathy along with neurological symptoms was clinically evocative of ADEM diagnosis.

As acute disseminated encephalomyelitis usually mimics viral or bacterial meningoencephalitis, a spinal tap for CSF analysis is systematically performed to exclude an infectious process. It may reveal inflammatory findings such as increased proteins rate up to 1.1g/l (found in 15%-60% of patients) and pleocytosis (high count of lymphocytes and monocytes) seen in 25%-65% of patients [4]. Whilst in pediatric patients with ADEM, CSF leukocytes count is normal in 42%-72% of cases [2]. Intrathecal oligoclonal bands are rarely found in patients with ADEM, out of 53 patients studied in 3 case series, intrathecal OCBs were detected in only 1 patient (1,9%) [1]. If detected, they are usually represented as mirror bands found in both serum and CSF, therefore they would not be considered as true OCBs, but rather suggest an antibody production that is not intrathecally limited [1].

In serum, multiple antibodies targeting myelin autoantigens have been identified. In particular, anti-myelin oligodendrocyte glycoprotein antibodies that have been reported to be present in up to 40% of pediatric ADEM cases [5]. The presence of these antibodies is an indicator of a monophasic course of ADEM [6], and a negative predictor of an eventual diagnosis of MS [7]. Anti-myelin oligodendrocyte glycoprotein antibodies are found exclusively in demyelinating disorders, which may help ruling out viral encephalitis [5].

Acute disseminated encephalomyelitis diagnosis is based on clinico-radiological findings. Brain MRI with gadolinium injection is the best-suited imaging exam to visualize demyelinating lesions. It shows multiple large areas of increased signal in T2-weighted and fluid attenuated inversion recovery sequences, poorly bordered, and asymmetrically implicating the cortical and central white matter, cortical gray-white matter junction, thalami, basal ganglia, cerebellum, and brainstem [1]. Existence of lesions enhancement in post-contrast T1-weighted sequence is highly variable between studies (10%-95% of cases), considered to be probably depending on the time brain MRI was performed in the disease course [8]. The major differential diagnosis of ADEM in pediatric patients is multiple sclerosis; distinctive brain MRI characteristics could help make the difference [4]. The features typically found in ADEM and missing in MS include deep gray matter and cortical involvement, bilateral and poorly marginated diffuse lesions. Whereas, other existing features in MS and absents in ADEM entail perpendicular lesions to long axis of corpus callosum (known as Dawson's fingers), and black holes in T1-weighted sequences [1]. Multiple sclerosis diagnosis is not made during the first event, but could be suggested in the presence of old and new lesions at the MRI, and meeting revised McDonald criteria [5].

In 2007, the International Pediatric Multiple Sclerosis Study Group (IPMSSG proposed diagnostic criteria for pediatric acquired demyelinating disorders of the CNS, including ADEM. In 2012, these criteria were revised and updated so that any other possible demyelinating disorder has been cautiously excluded before establishing ADEM diagnosis.

Thence, the required criteria for pediatric ADEM diagnosis [9]:

1. A first multifocal, clinical CNS event with presumed inflammatory demyelinating cause.
2. Encephalopathy that cannot be explained by fever.
3. Brain MRI is abnormal during the acute (3 month) phase (*typically shows diffuse, poorly demarcated, large, >1-2 cm lesions involving predominantly the cerebral white matter; T1 hypo intense white matter lesions are rare. Deep grey matter lesions (eg, thalamus or basal ganglia can be present).*
4. No new clinical and MRI findings emerge 3 months or more after the disease onset.

Another differential inflammatory and demyelinating disorder correspond to clinical isolated syndrome. It is considered at the onset of monofocal or multifocal neurological symptoms and the absence of encephalopathy [5]. Clinical isolated syndrome include optic neuritis, transverse myelitis, hemiparesis, monoparesis, and brainstem syndromes [9]. When optica neuritis and myelitis are predominantly presented, neuromyelitis optica diagnosis should be suggested, the presence of anti- aquaporin-4 antibodies is highly evocative of it and could rule out ADEM diagnosis [9].

Acute disseminated encephalomyelitis treatment lie on high-doses of intravenous corticosteroids as a first line therapy [5], the recommended regimen consists of methylprednisolone perfusion at the dose of 20-30 mg/kg/d (maximum dose of 1000 mg/d) for 5 days, followed by an oral intake of prednisone tapered over the period of 4-6 weeks with a starting dose of 1-2 mg/kg/d [1]. Full recovery has been reported in 60%-85% of cases [3]. Although, a period less than 3 weeks of steroid tapering might increase the risk of relapses [1].

Intravenous immunoglobulin perfusion comes as second-line therapy in steroids unresponsive ADEM patients or in case of contraindication of corticosteroids [5]. The total dose is 2 g/kg administered over the period of 2-5 days [1]. Treatment with intravenous immunoglobulin has proven effective in about 40%-50% of steroid-resistant patients [4].

Plasma exchange, also known as plasmapheresis, is usually recommended for therapy-refractory patients with fulminant disease course, its regimen consists of 5-7 exchanges and the reported efficacy goes up to 40% [4]. Nonetheless, it has recurrent complications that include anemia, hypotension, hypocalcemia, thrombosis, and infections [3].

In pediatric ADEM, the prognosis is quite favorable in 60%-85% of cases, patients usually start recovering from their neurological deficits within days after treatment start, with complete recovery few weeks later [1]. Residual severe disability is rare, reported in only 7% of children with ADEM [4].

## Conclusion

Acute disseminated encephalomyelitis is an inflammatory demyelinating disease of the CNS that usually ensues an infectious event or a vaccination, manifested clinically with an encephalopathy and neurological deficits, its diagnosis is based on clinico-radiological findings. ADEM carries a good prognosis when treated with immunosuppressive agents, which is the case of our patient that showed a fast recovery when he was put on high dose of corticosteroids.

## Patient consent

Written consent was given by the patient's mother for publishing this case report.
